# Pilot Acute Safety Evaluation of Innocell™ Cancer Immunotherapy in Canine Subjects

**DOI:** 10.1155/2020/7142375

**Published:** 2020-10-08

**Authors:** Raymond P. Goodrich, Jon Weston, Lindsay Hartson, Lynn Griffin, Amanda Guth

**Affiliations:** ^1^Colorado State University, Fort Collins, CO, USA; ^2^PhotonPharma, Inc., Fort Collins, CO, USA

## Abstract

**Background:**

We are developing cancer immunotherapy based on the use of autologous tumor tissue that has been rendered replication-incompetent but maintains phenotype and metabolic activity post-preparation.

**Aim:**

The aim of this study was to evaluate safety and tolerance to injection of the inactivated tumor cell and adjuvant preparation (Innocell™) within 24 hours of administration in a pilot study in canine patients with solid organ tumors. *Methodology*. Three canine patients demonstrating accessible solid organ tumors of various types were assessed in this study. The local site injection was monitored post-treatment. Clinical signs of adverse reactions were monitored for 24 hours post-treatment. Blood samples were taken pre-treatment and at 8 and 24 hours post-treatment for all subjects. One subject provided samples at 7 days post-treatment. All blood samples were analyzed for cytokine content for both immune system-associated and tumor-associated cytokines.

**Results:**

No signs of adverse reactions at the site of injection or systemically were observed in the study period. A slight fever and lethargy were reported in one subject by the owner post-vaccination. Immune system-associated cytokine levels in two of the three animals were elevated post-treatment. Tumor-associated cytokine levels in all three subjects declined post-treatment from baseline levels with the effect most prominent in the subject with a non-excised tumor.

**Conclusion:**

Subcutaneous injection of the inactivated tumor cells and adjuvant was well tolerated in this pilot study. Cytokine responses observed were in line with the intended use of the treatment in stimulating immune response without adverse clinical observations. Additional evaluation is warranted.

## 1. Introduction

We are developing and have previously reported on the behavior of a new cancer immunotherapy approach for treating solid organ tumors in humans. This method (Innocell™) relies on the use of autologous tumor tissue to provide antigen presentation to the immune system following treatment of the cells with Riboflavin and UV light, which prevents cell replication while maintaining tumor cell metabolic function and phenotypic integrity [1]. Initial studies in mice demonstrated the ability of this approach to suppress tumor cell growth, generate dendritic and T cell activation, slow metastatic disease progression, and induce production of an immune response in murine tumors for breast cancer when used in combination with adjuvant [1].

Cell-based immune therapies have recently become an important therapeutic approach for dealing with certain forms of cancer. Treatment of solid organ tumors has been problematic. Many of these approaches target generic antigens, which in some cases are commonly shared between healthy and cancerous cells. The approach used in elimination of B-cell populations in haematologic neoplasias using chimeric-antigen receptor (CAR) T cell therapies would not be possible with tumors involving organs such as the lung, pancreas, and liver.

Many groups have been evaluating the use of cancer vaccines for the stimulation of immune response mechanisms against the tumor in an autologous setting [2–4]. We have utilized an approach using autologous tumor tissue, in combination with adjuvant, to generate an immune response against solid organ tumors and have previously reported on its performance in several murine models. The preliminary data generated in those studies indicated that RF+UV inactivation prevented tumor cell proliferation of both mouse and human tumor cells and prevented growth of the RF+UV-inactivated tumor cells in vivo, while maintaining protein expression. We also observed increased DC maturation and IFN-*γ* production in spleen cells for mice vaccinated with either inactivated tumor cells only or the Innocell™ vaccine (and restimulated with RF+UV-inactivated 4T1 cells in culture).

Mouse models represent an important first step in the evaluation of these technologies in the characterization of the immune response. The next step in development is often the evaluation of performance in an autochthonous tumor setting in animals [5]. This study was designed to test the safety of this autologous cancer cell vaccine (Innocell™) in client-owned dogs with spontaneous cancers. The utilization of canine patients for evaluation of human therapeutic approaches in cancer therapy has been well documented [6, 7].

## 2. Materials and Methods

### 2.1. Study Design and Animal Use Committee Approvals

Dogs with easily accessible tumors allowing the isolation of biopsy material or excised tumor tissue were enrolled in a veterinary clinical study under an approved study protocol at the Flint Animal Cancer Research Center at Colorado State University. The study protocol was reviewed by the University Institutional Animal Care and Use Committee (IACUC) prior to enrollment, and informed consent from all animal owners was received prior to inclusion in the study.

The study protocol included analysis of local injection site responses by veterinary medicine trained staff as well as systemic monitoring of clinical signs, imaging, and blood draws for cytokine signals following injection and for a period of up to 24 hours posttreatment. All reports, imaging, blood sample, and clinical measurements were performed by veterinary medical staff at the Flint Animal Cancer Research Center, independent of the study sponsor.

### 2.2. Production of Vaccine Material

**Figure 1:**
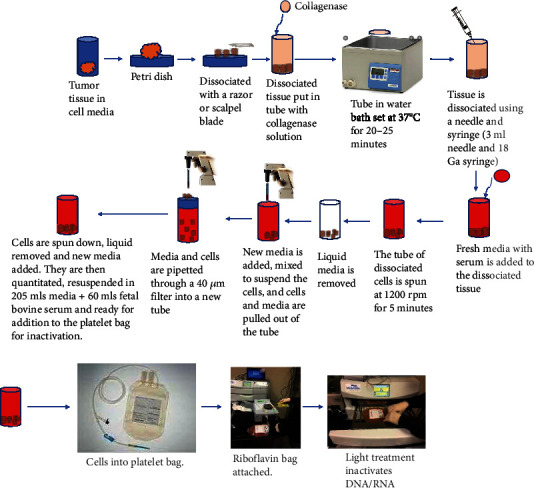
Vaccine production process outline. These diagrams show the steps going from isolation of the solid organ tumor to preparation for inactivation of the cells in the illumination device. Disposables and equipment utilized for the process come from products suitable and marketed for human use in standard blood banking operations.

The overall processing steps for the production of the Innocell™ vaccine are described in Figure 1. Biopsied or surgically removed tumor cells were processed on the day of isolation into cell suspensions and inactivated using a photochemical process as described previously [8]. Briefly, tumor cell material was minced and then mixed with collagenase and manually manipulated until an even cell suspension was produced using an 18-gauge syringe and needle. This suspension was spun down, and the supernatant was removed. The concentrated cells were counted using a brightfield cell counter (Nexcelom) and resuspended in media (MEM+20% FBS) to a volume of 265 mls. 35 mls of a 500-micromolar Riboflavin solution in saline (Terumo BCT, Lakewood, CO) was added to this mixture. The mixture was placed in a citrate plasticized PVC bag and illuminated with UV light at a total energy dose of 300 joules (Mirasol PRT device, Terumo BCT, Lakewood, CO).

### 2.3. Innocell Administration

Following inactivation of the tumor cells, the samples were concentrated by centrifugation and resuspended in media (MEM+20% FBS). For Subject One, cells were divided into 1.5 × 10^7^ cells per vial and were frozen in serum-free cell media (ATCC). For Subject Two, cells were frozen prior to inactivation. After thawing, cells were inactivated as described above, then washed three times in HBSS and counted prior to the addition of adjuvant. For Subject Three, samples were stored for 24 hours at +4°C prior to administration. Prior to vaccine administration, 500 micrograms of the adjuvant, CpG ODN 1668 (Enzo), was added in 500 microliters of PBS and 1500 microliters of inactivated cells in HBSS. Dogs were injected intradermally in 4 loci for lymph nodes, two (each of the forelimbs) and two (each of the hind limbs) of each animal [9].

### 2.4. Cell Counting of Vaccine Preparations

Cells obtained from tumor tissue were quantitated using brightfield imaging (Nexcelom). On average, each vaccine contained 1-2 × 10^6^ cells. From Subjects One and Two, we obtained 834 mg and 1.02 g of tumor tissue and obtained yields of 5.0 × 10^7^ and 5.4 × 10^7^ cells, respectively. Subject Three provided 400 mg of tissue. A cell count was not performed on Subject Three. Prior internal studies yielded values on average of 1.0 × 10^7^ tumor cells per gram of tissue (data not shown), consistent with published observations of yields in this range [10].

### 2.5. Clinical Monitoring

Dogs were monitored overnight for any adverse vaccine reactions. Injection sites were evaluated post-vaccination at 15 minutes, 30 minutes, 1 hour, 8 hours, and 24 hours. Blood was collected at 0 hours (pre-vaccination) and 8 hours and 24 hours post-vaccination and was analyzed for any changes above the baseline levels following vaccination. In one animal (Subject One), additional follow-up was performed on Day 7 post-treatment.

### 2.6. Cytokine Measurements

Cytokine responses were measured using a bead-based pre-mixed Multiplex assay (Sigma Aldrich) and corresponding plate reader (MAGPIX, Luminex Corp.). A panel of 13 cytokines was measured prior to treatment, 8 hours post, 24 hours post, and on Day 7 for one subject (Subject One).

## 3. Results

### 3.1. Clinical History and Observations

No significant observations of safety concern or intolerance to the vaccine administration were observed over the 24 hours post-injection in any of the subjects in the study. Individual details for each study subject were reported as follows.

#### 3.1.1. Subject One

Subject One was an 11-year-old female Labradoodle mix and was diagnosed with a hepatocellular carcinoma mass. The tumor was excised leaving indistinct margins as determined by histopathology performed on the excised tumor material. A two-week recovery period post-surgery prior to vaccination was utilized to allow clear distinction in response to the treatment and to separate from the effects of surgery. No adverse events were recorded following vaccination. At all post-vaccination time points, injection sites were rated “good” with no redness, swelling, or abnormalities noted. In addition to zero-, 8-, and 24-hour blood draws, Subject One had an additional draw after one week. At the one-week checkup after vaccination, the owner reported that Subject One had displayed lethargy and elevated temperature for a few days following vaccination.

At initial presentation, ultrasound imaging and biopsy of Subject One showed a large lobulated mass with heterogenous echogenicity in the left aspect of the liver. This mass measured at least 6.7 × 6.1 × 4.4 cm. Multiple other hypoechoic nodules were observed throughout the liver, which had diffusely hyperechoic parenchyma. Observations were consistent with hepatocellular carcinoma with concurrent metastatic disease or comorbid process such as hepatocellular vacuolization or nodular hyperplasia.

**Figure 2:**
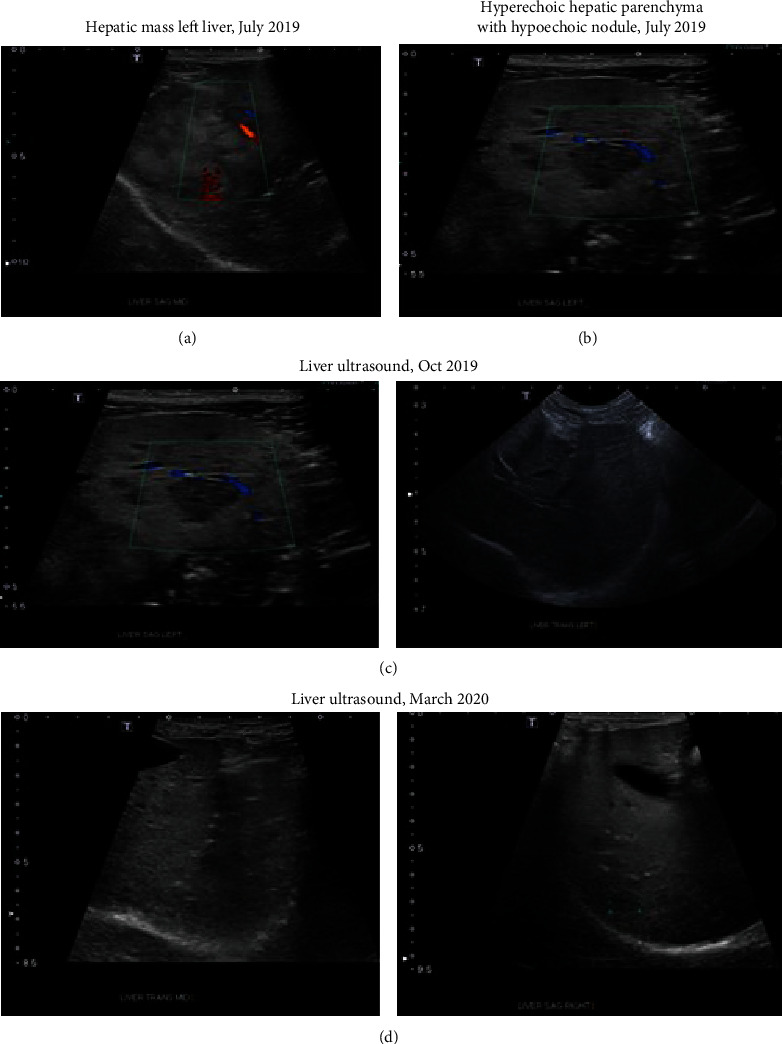
Ultrasound images of Subject One with hepatocellular carcinoma. (a, b) Presurgical images of liver tumor. (c) 3-month image posttreatment of the resected area. (d) 7-month image posttreatment of the resected area. No signs of tumor regrowth or metastases were observed.

An ultrasound taken 3 weeks post initial presentation showed that the previously identified hepatic mass in the dorsal mid to left aspect of the liver remained similar in size (6.5 × 6.1 × 6.9 cm) but was more heterogenous in appearance (Figures 2(a) and 2(b)). Doppler interrogation showed blood vessels extending from the liver into the mass with mild internal signal/vascularity. There continued to be other hypoechoic nodules throughout the remainder of the liver, which was diffusely hyperechoic. The liver itself was subjectively enlarged with rounded borders that extended beyond the stomach. Results of this analysis and sample aspirate showed that the hypoechoic nodules represented hepatocellular vacuolization.

Three months following lobectomy and treatment with the Innocell™ vaccine, ultrasound imaging showed the absence of the previously described hepatic mass secondary to the known liver lobectomy (Figure 2(c)). The remainder of the liver continued to have mildly, diffusely hyperechoic to heterogenous parenchyma with multiple well-defined hypoechoic nodules measuring up to 15.1 mm in diameter seen throughout. A fine needle aspirate of these nodules was performed during this study and was absent of evidence of metastasis.

At seven months post-treatment, there continued to be a subjectively decreased amount of hepatic parenchyma on the left aspect of the liver, consistent with the previous lobectomy. The prior left liver lobectomy showed no evidence of recurrent disease (Figure 2(d)). Observations of the hepatic parenchyma (heterogenous and hyperechoic with multiple hypoechoic nodules) were likely due to a benign process given timeline. Differentials included hepatocellular vacuolization or nodular hyperplasia. Neoplastic disease was considered unlikely.

#### 3.1.2. Subject Two

Subject Two was a 9-year-old, female spayed, German Shorthaired Pointer diagnosed with a cutaneous mast cell tumor. Subject Two also had an additional right lateral flank mass with cytology consistent with a lipoma. The mast cell tumor was excised with narrow margins (microscopic tendrils extending away from the mass remained; residual gross disease remained). This subject received Innocell™ treatment two weeks following surgery and removal of the tumor material.

Subject Two was reported to be receiving steroids (prednisolone 20 mg tab, P.O. (oral administration), Q.D. (once daily)) plus an antihistamine (diphenhydramine, 25 mg every 8 hours) to control histamine overload associated with mast cell tumors. At all time points, injection sites were rated “good” with no redness, swelling, or abnormalities. No adverse events were reported in the 24 hours following treatment.

#### 3.1.3. Subject Three

Subject Three was a 13-year-old, female spayed Chihuahua, diagnosed with soft tissue sarcoma. An initial incisional biopsy of the tumor was made following study enrollment. The subject received Innocell™ treatment on the following day using material isolated from the biopsy. At each examination time point, injection sites were rated “good” with no redness, swelling, or abnormalities. No adverse events were reported in the 24 hours following treatment. Subsequently, Subject Three started palliative radiation therapy two days after treatment with Innocell™.

### 3.2. Immune Response Profiles in Treated Subjects as Measured by Cytokine Levels

**Figure 3:**
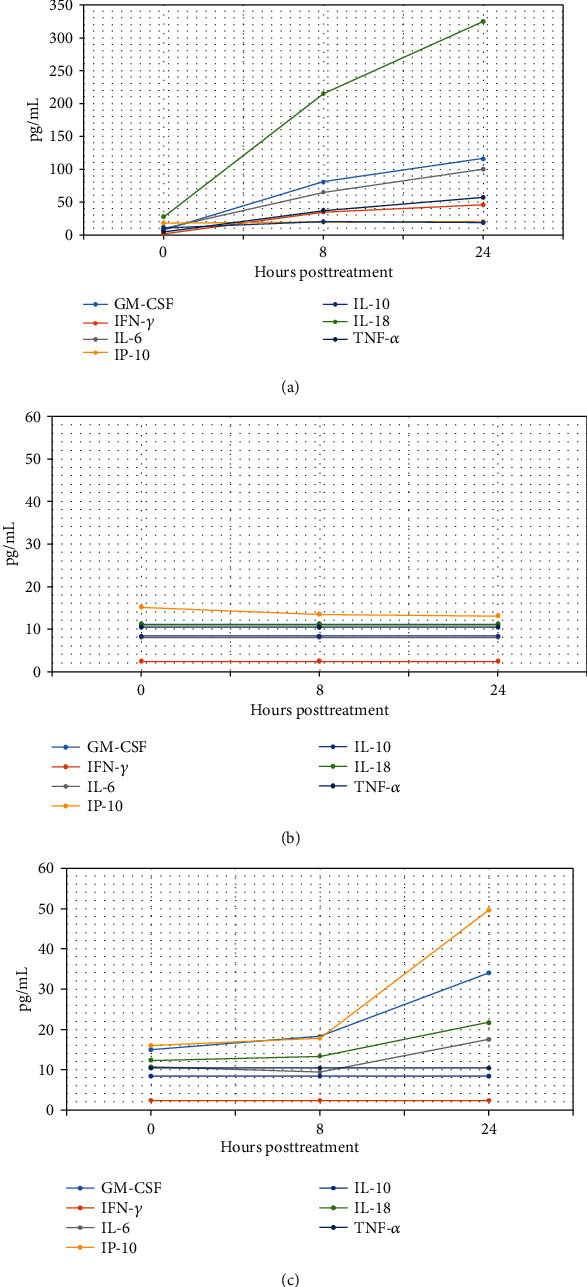
(a) Cytokine profile in Subject One, (b) cytokine profile in Subject Two, and (c) cytokine profile in Subject Three.

Cytokine profiles observed in all subjects following treatment are recorded in Table 1 and displayed in Figure 3. Subject One (Figure 3(a)) showed notable increases in granulocyte-macrophage colony-stimulating factor (GM-CSF), which generally stimulates monocytes and neutrophils and reduces the risk for febrile neutropenia in cancer patients [11]. It is also known to induce the differentiation of myeloid dendritic cells (DCs) that promote the development of T-helper type 1 (cellular) immune responses. Likewise, Interferon-gamma (IFN-*γ*) was increased in Subject One. This cytokine is normally secreted by activated T cells and natural killer (NK) cells and promotes macrophage activation, enhances antigen presentation, orchestrates activation of the innate immune system, and controls cellular proliferation and apoptosis [12].

Subject One also demonstrated increased levels of Interleukin-6 (IL-6), Interleukin-15 (IL-15), Interferon gamma-induced protein 10 (IP-10), Interleukin-10 (IL-10), Interleukin-18 (IL-18), and tumor necrosis factor-alpha (TNF-*α*). Each of these increases is associated with a cytokine that is normally involved in signaling mechanisms to promote immune system responses via the innate and adaptive immune systems [13]. Levels for these agents decreased but remained elevated above pretreatment baseline levels for a period of 7 days post-treatment. In contrast for Subject One, tumor-associated cytokines such as Interleukin-8 (IL-8) and monocyte chemotactic protein-1 (MCP-1) declined over the study period from pre-treatment (two weeks post-resection) levels [14].

Subject Two (Figure 3(b)) displayed limited immunoinflammatory cytokine responses, likely due to concomitant administration of steroid and antihistamine, which are known to suppress immune responses and inflammation. The tumor-associated cytokines did show a reduction in amounts detected after treatment, suggestive of primary tumor activity reduction from baseline. The magnitude of this response may also have been muted, however, by the presence of a second untreated lipoma present in the same subject. Decreases in tumor-associated cytokines indicative of a reduction in activity of tumors were observed in Subject Two for IL-8 and MCP-1. These levels also remained below baseline values observed pre-treatment.

With the exception of TNF-*α* and IFN-*γ*, a similar response of immune-associated cytokines was observed for Subject Three (Figure 3(c)) as were noted in Subject One. For Subject Three, levels of GM-CSF, IL-6, IL-15, IP-10, and IL-18 all increased above the baseline by 24 hours posttreatment. Subject Three also displayed 40%-60% decreases in tumor-associated cytokines (MCP-1 and IL-8) from 8 to 24 hours after treatment.

**Figure 4:**
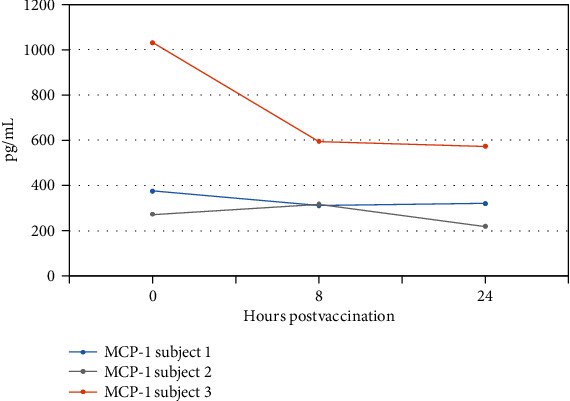
MCP-1 response post-vaccination.

Subject Three was the only one of the three dogs to have a biopsy rather than tumor resection. Significantly, in absolute numbers, Subject Three showed the largest decline in tumor-associated cytokines IL-8 and MCP-1 (Figure 4). This could be due to the fact that the whole tumor remained in situ at time of treatment, whereas the other dogs had the tumors removed two weeks prior to treatment and thus had already seen a decline in these cytokines due to removal of the tumor pre-treatment.

## 4. Discussion

The canine subjects in the trial displayed three very different cancer scenarios in terms of types of cancers, tumor resection versus biopsy, burden of additional cancers, and concomitant drug therapies. The trial demonstrated no significant 24-hour safety events following treatment of dogs with Innocell™ in the format intended for human subject use. No severe adverse events were observed post-treatment, and the injection sites proved unremarkable in terms of local inflammation.

Cytokine reactions indicative of an immune mobilization response, the goal of the intended treatment, were clear in two of three dogs. The low response in Subject Two could be explained by the effects of concomitant steroid and antihistamine therapies.

All three dogs showed tumor-associated cytokine reduction following treatment. The extent of this reduction varied among the animals, likely based on the presence or absence of a primary tumor and the presence or absence of additional tumors.

Limitations of this work include the fact that by design, acute safety trials generally only allow a follow-up for 24 hours of evaluation posttreatment. Longer-term outcomes for all three subjects were not available. However, the owner of Subject One did voluntarily provide longer-term follow-up information. At seven months post-treatment, Subject One showed no signs of tumors, tumor regrowth, or tumor metastases even though the surgical excision left indistinct margins with potential cancerous tissue left behind (see Figure 2).

Mouse models are often the first step in the development of a new vaccine or therapeutic candidates [15]. The availability of analytical reagents suitable for the evaluation of immune response for both cellular and humoral pathways makes murine models preferable for early reads in efficacy. The limitation of such models, however, is that tumors are usually artificially induced or implanted and thus do not represent spontaneously generated tumors that occur naturally. Canine models have been used increasingly to bridge the gap between murine studies and human clinical evaluation because they afford a way to study naturally occurring tumors that mimic the clinical situation in the human setting [16, 17]. A limitation of canine models, however, is the availability of analytical reagents that allow extensive evaluation of immune response at the cellular level [17]. In this work, we utilized acute cytokine response levels as a measure of subject response to vaccination. This approach is limited in terms of an efficacy measure of vaccine performance. Despite these limitations, however, the trial results suggest evidence of acute safety and tolerance of the proposed administration route and dosing that is being proposed for the Innocell™ product in human clinical research. This study also provides additional data to bridge the gap between murine model studies and human clinical trials.

## Figures and Tables

**Table 1:** Results from cytokine analysis of blood samples from three study subjects at various time intervals postadministration of Innocell product. Results include data for immune system-related (GM-CSF, IFN-*γ*, IL-6, IL-8, IL-15, IP-10, IL-10, TNF-*α*, and IL-18) and tumor-related (IL-8 and MCP-1) cytokines.

	Time posttreatment (hours)	GM-CSF (pg/ml)	IFN-*γ* (pg/ml)	IL-6 (pg/ml)	IL-8 (pg/ml)	IL-15 (pg/ml)	IP-10 (pg/ml)	IL-10 (pg/ml)	IL-18 (pg/ml)	MCP-1 (pg/ml)	TNF-*α* (pg/ml)
Subject One	0	7.75	1.45	9.67	7859	53.78	18.25	10.89	27.35	374.58	4.8
	8	80.96	34.58	65.02	4168	685.67	19.87	20.09	214.6	309.94	37.06
	24	116.18	46.13	100.22	2882	967.45	20.68	18.67	324.23	319.76	56.88
	168	50.22	24.71	42.94	2516	525.18	17.44	10.16	138.95	167.86	23.87

Subject Two	0	≤11.1	≤2.37	≤8.02	4105	6.96	15.13	≤8.34	≤10.96	271.7	≤10.39
	8	≤11.1	≤2.37	≤8.02	3559	8.32	13.41	≤8.34	≤10.96	316.29	≤10.39
	24	≤11.1	≤2.37	≤8.02	3655	2.83	13.01	≤8.34	≤10.96	217.96	≤10.39

Subject Three	0	14.98	≤2.37	10.66	16957	31.59	15.92	≤8.37	12.29	1032	≤10.39
	8	18.29	≤2.37	9.35	7419	14.3	17.74	≤8.37	13.29	593.99	≤10.39
	24	34.04	≤2.37	17.56	11631	46.63	49.6	≤8.37	21.71	571.2	≤10.39

Values listed as ≤ indicate values below the limit of detection.

## Data Availability

All relevant data are within the manuscript and its supporting information files. All data are fully available without restriction.
